# Another perspective: Reflections on using qualitative video-recall procedures in sexual communication research with partnered gay men

**DOI:** 10.1177/13634607211000203

**Published:** 2021-03-04

**Authors:** Mathew R Gendron, Sharalyn Jordan

**Affiliations:** The 8166 University of British Columbia, Canada; 1763 Simon Fraser University, Canada

**Keywords:** Gay male relationships, sexual communication, qualitative, video-recall

## Abstract

As sex cultures evolve in complexity, so too must our research procedures. We introduce qualitative video-recall procedures and discuss the unique opportunities they present in the study of sexual communication. In a pilot study, three diverse gay male couples had video-recorded conversations about aspects of their sexual relationships that they wished to change or explore. Partners then individually watched and reflected on their partnered conversations during open-ended video-recall interviews. We discuss how reflexively engaging with these research procedures enabled the first author to (1) confront dominant and restrictive assumptions about partnered sex, (2) observe how interpersonal dynamics shape sexual communication, and (3) enhance cultural reflexivity.

Throughout history, social and political discourse has shaped how people navigate sex and sexuality. Although undoubtedly apparent in all sexual relationships, these connections are particularly salient for individuals of non-dominant genders and sexualities. As demands for social, economic, and health protections intersect with a rise in conservatism in the West, contemporary discourses offer paradoxical messages about queer sex. Amidst divisive political rhetoric and massively lethal anti-queer violence (Edelman, 2018), queer Americans have recently secured protection against discrimination in the workplace under the Civil Rights Act. Similarly, queer Canadians are marrying with increasing frequency (Statistics Canada, 2017), while advocates continue to push for federal protection against conversion therapy (House of Commons, 2019). In order to keep up with the varied ways that these sociopolitical shifts materialize in queer peoples’ sex lives, researchers must innovate creative approaches to the study of sex and sexuality.

We introduce qualitative video-recall procedures as one such approach. Drawing from our work on a pilot study with three diverse gay male couples, we explore the reflexive value of using these procedures to study queer couples’ sexual communication. Our purposes for this paper are threefold. First, we aim to demonstrate how these procedures enable researchers to confront restrictive assumptions about partnered sex. Second, we aim to highlight how these procedures help researchers attend more deeply to interpersonal dynamics that inform conversations about sex. Finally, we aim to show how these procedures enhance cultural reflexive practices. Using data from this pilot project, we reflect on the exciting promise that these procedures hold. Although we attend primarily to concerns of method, we hope the novel empirical knowledge produced through the pilot also inspires additional lines of research in the study of gay men’s sexual communication.

## Video-recall procedures

Technological advancements of the 1980s allowed relationship researchers to study communication processes in innovative ways (Welsh and Dickson, 2005). Video-recall procedures were developed to capture couples’ subjective experiences during partnered communication. Using these procedures, researchers invite partners to engage in self-directed, video-recorded conversations about a given topic. Researchers then instruct partners to separately view these video-recorded conversations and reflect on their experiences at the time of the conversation (Welsh and Dickson, 2005). The development of these innovative procedures has enabled researchers to attend to couples’ subjective, in vivo experiences during partnered communication.

## Quantitative video-recall

Relationship researchers have almost solely used quantitative approaches to video-recall. Most often, partners are asked to provide continuous numerical ratings of their affective experiences while viewing their video-recorded conversations (e.g., Gottman et al., 2003). In their seminal study using video-recall procedures, Levenson and Gottman (1983) asked participants to manipulate a dial to rate their affect on a scale that ranged from very negative to very positive. Couples were instructed to rate their affect based on how they recalled feeling at the time of the conversation, as opposed to their experience while reviewing the conversation (Gottman and Levenson, 1985; Levenson and Gottman, 1983). The perceived ability to have direct access to participants’ subjective experiences encouraged a proliferation of studies that used quantitative video-recall procedures to study couples’ communication (Powers et al., 1994).

Despite attending to partners’ subjective experiences in innovative ways, these quantitative video-recall procedures lack the ability to generate previously unknown qualities of partnered communication. Quantitative video-recall procedures are used to test hypotheses that have been developed based on pre-existing knowledge about intimate relationships (Welsh and Dickson, 2005). Because this knowledge is largely informed by empirical understandings of conflict and support conversations between heterosexual partners (Kurdek, 2005), these approaches restrict researchers from exploring other topics (e.g., sex) and relationships (e.g., queer partnerships).

## Qualitative video-recall

By contrast, qualitative video-recall procedures enable researchers to generate novel understandings about how diverse couples talk about sex. These procedures are similar to the quantitative approaches discussed earlier; however, instead of numerically rating their experience on a set of predetermined measures, participants are invited to engage in reflective dialogue through open-ended interviews while separately viewing their recorded coupled interaction (Kagan et al., 1963). During these interviews, researchers elicit reflections from each partner as they separately view their video-recorded partnered conversation (Young et al., 1994). Videos are periodically paused, and partners are asked open-ended questions about their experience during their partnered interactions. These interviews’ semi-structured approach enables researchers to explore areas that hold relevance for their participants, thereby constructing novel understandings of partnered communication.

One of the most widely used forms of qualitative video-recall, interpersonal process recall (IPR) was originally developed as a clinical training tool in the field of Counselling Psychology (Kagan et al., 1963). Since its development, IPR has been used to produce rich, reflective interview data that are then analyzed through a variety of approaches (Rennie, 1992). Whereas IPR does not adhere to a single approach to qualitative data analysis, the more recently developed action-project method (APM) analyzes video-recall data through contextual action theory (Young and Valach, 2016; Young et al., 2005). Consistent with this specific theory of action, the APM uses qualitative video-recall procedures to explore how dyads co-construct and achieve joint goals through action, including dyadic communication. Although qualitative video-recall procedures have mostly been used to study clients’ experiences in therapy (e.g., Rennie, 1992) and parent–child interactions (e.g., Young et al., 2001), more recent studies have used these procedures to explore partnered interactions in intimate relationships (Domene et al., 2012). Grounded in constructionist philosophies, these procedures hold particular promise for developing contextualized understandings of sexual communication.

## Applying qualitative video-recall procedures to partnered gay men’s sexual communication

Despite qualitative video-recall procedures’ strong potential for deepening understanding of various forms of partnered communication, this pilot is the first known study to use these procedures to explore partnered sexual communication. This project was conducted as part of the first author’s graduate research program in Counselling Psychology, for which the second author served as senior supervisor. In response to the literature’s disproportionate investment in understanding gay male sexual communication through sexual health frameworks (e.g., Gomez et al., 2012; Mitchell, 2014; Prestage et al., 2006), we used qualitative video-recall procedures to explore partnered gay men’s sexual communication processes. Through using these robust research procedures, we aimed to build upon the limited available observational research with gay male couples (e.g., Gottman et al., 2003) to more fully understand how partnered gay men talk about sex. The pilot was guided by the following broad research questions: (1) How do partnered gay men communicate about sex? and (2) How do partnered gay men use sexual communication to establish and to maintain pleasure and intimacy in their relationships?

## Data collection and analysis

After attaining institutional ethics approval for research involving human participants, three gay male couples were recruited through advertisements posted by local queer community organizations that partnered with the study. We used purposive sampling to select three couples from the larger sample of eligible and interested participants. We selected these couples because they represented diversity in age, relationship status, relationship length, and ethnicity that we believed would serve the exploratory purposes of this pilot project. The sample included racial and ethnic diversity within and between couples, including three White Euro-Canadians, two Southeast Asian-Canadians, and one self-identified Filipino-Canadian. Selected participants were 20 to 46 years old and included one transgender man and five cisgender men. All selected couples identified their partnerships as committed, ranging from two and a half years to seven years in length. Couples varied in how they structured monogamy in their relationships, ranging from monogamous to polyamorous. Once screened for eligibility, couples scheduled sessions at a university campus in a major Canadian city. During these sessions, eligible couples were asked to have two video-recorded conversations about their relationship, followed by separate video-recall interviews.

## Collecting conversation data

Following a warm-up conversation about how they met, couples had 15-minute video-recorded conversations about something they wanted to change or explore in their sexual relationships. Before engaging in these conversations, partners were separated and paired with a member of the research team, both of whom were gay-identified men. In separate rooms, partners were asked to compile lists of topics that they would and would not feel comfortable discussing with their partner during their session. Once completed, the research team met privately to compare lists. Couples were then presented with a list of their overlapping approved topics and partners collaboratively selected which topic they would discuss. This topic selection procedure was practiced to ensure that partners did not feel coerced into speaking about topics that they were not comfortable discussing in a research setting. Couples then discussed their chosen topics during self-directed 15-minute conversations.

## Collecting video-recall data

Following these conversations, partners were paired with a member of the research team to separately review their video-recorded conversations. During these video-recall interviews, partners and researchers paused the video recordings at approximately 1-minute intervals and partners were asked to reflect on their thoughts, feelings, or motivations at that moment in the conversation. This semi-structured approach to video-recall enabled participants and researchers to explore particularly salient aspects of couples’ conversations (Welsh and Dickson, 2005).

Given the variability of this open-ended approach, partners did not necessarily reflect on the same moments of conversation, nor were they necessarily asked to engage in the same reflective processes. Researchers guided partners’ reflections using predetermined open-ended prompts informed by the APM (e.g., “How did it feel to hear your partner say that?”) and with process observations informed by IPR (e.g., “Tell me about what was going on for you when you rolled your eyes”). In addition to these prompts, participants also initiated reflections by elaborating on the conversation’s content or on their experience at the time of conversation. Through this collaborative approach to video-recall, partners were empowered to take ownership of their stories and highlight moments that they deemed particularly important. These video-recall interviews lasted approximately 1 hour, after which partners debriefed their participation, completed video release forms, indicated a charity they wished to donate to in lieu of direct compensation, and provided pseudonyms to represent their participation in the study.

## Data analysis

Following these sessions, I (MG) transcribed couples’ partnered conversations and video-recall interviews. Informed by Saldaña's (2013) eclectic coding method, I then analyzed the transcripts three times, coding different features with each reading. During the first reading, I generated open-ended codes to succinctly capture the content of couples’ partnered conversations and separate video-recall interviews. In the second reading, I attended to the discursive processes couples used to navigate dominant sexual scripts (Frith and Kitzinger, 2001; Simon and Gagnon, 1986). Finally, I attended to affect during the third reading by generating codes that captured couples’ verbal and non-verbal behaviors during their partnered conversations. I then conducted a thematic analysis on these generated codes. I consulted with the second author and with a colleague who is experienced in observational couples’ communication research during this process.

## Member checking process

Approximately nine months after their sessions, participants were invited to provide feedback and to contribute to these preliminary analyses. After explaining the review process by phone, I individually emailed partners excerpts that referenced their participation (i.e., their partnered conversation and their own video-recall interview). Participants were prompted to provide open-ended feedback about these interpretations. If participants believed that they would be identifiable to readers, they were asked to offer changes that would further protect their anonymity. Participants were also prompted to report whether they felt comfortable with the selected quotes. Finally, participants were asked to note any potential factual errors in the manuscript (e.g., if I misreported their age). None of the participants provided alternative analyses and one participant provided additional contextual information, which was subsequently added to the analyses.

## Discussion

In the following section, I (MG) review three pieces of learning that surfaced while using qualitative video-recall procedures during this project with partnered gay men. Organized as independent cases, I highlight critical teachings that emerged through engaging with each couple. I discuss how these procedures enabled me to (1) confront dominant and restrictive assumptions about partnered sex, (2) observe how interpersonal dynamics shape sexual communication, and (3) enhance my cultural reflexivity.

## Winchester and Russell: Confronting assumptions about partnered sex

By observing partners in dialogue, I was able to confront learned assumptions about sex. Educated in the fields of Psychology and Sexology, my early teachings about human sexuality relied heavily on cognitivist assumptions that reify sex and desire as relatively stable and measurable constructs. This project importantly stretched these theoretical assumptions to more fully consider how social and political discourses shape partnered sex. Having the unique opportunity to observe partners in dialogue and explore their discursive processes through video-recall interviews invaluably challenged these dominant sexual assumptions.

Twenty years ago, Frith and Kitzinger (2001) cautioned against cognitivism’s restrictive influence on the sociological study of sex. In their discussion of Simon and Gagnon’s (1986) sexual script theory, these authors noted how contemporary applications have rested upon individualistic assumptions that compromise the theory’s initial aim to situate sex in social and cultural context. I carried these theoretical tensions with me as I applied sexual script theory to the video-recall data I produced. Unsure of the extent to which sex relied on culture, I considered both cognitivist and discursive extensions of sexual script theory in my analytic approach.

Observing key moments in participants’ conversations, I came to appreciate how these conversations about sex were emergent and discursively produced. In resonance with Frith and Kitzinger’s (2001) discursive approach to sexual script theory, I was able to observe how sexual behavior became scripted through the ways that some couples discussed sex. By distinctly positioning themselves to hetero/cisnormative demands, Winchester and Russell scripted sex in ways that subverted dominant expectations and created novel opportunities for pleasure and intimacy. In addition to demonstrating how gay men use sexual scripting to destabilize stifling sexual expectations, these qualitative video-recall procedures also enabled me to reflect more deeply on ways in which individualistic assumptions pervade empirical understandings of sex.

## Exploring pornography through sexual scripting

During their partnered conversation, Winchester and Russell resisted cultural norms that encourage romantic partners to hide their pornography use (Rasmussen, 2016). They subverted this expectation for secrecy by openly discussing the possibility of viewing pornography together as a couple. During their conversation, both partners expressed shame and embarrassment regarding the type of pornography they watch and the amount that they view.

R: I don’t know, I’m self-conscious with the kind of porn that I watch, and like, will you like it, or will I be judged.

W: Yeah, totally, me too, me too. Just like, will I be judged, and yeah, totally.

R: But I know we wouldn’t. And it’s funny because I always clear the browsing history, ‘cause I’m like, self-conscious. Well, not so much about the type of porn I’m watching, it’s just like, “oh my God what if he sees how many sites that I’ve looked at,” or whatever.

W: (laughs)

R: I know, I know it’s silly. But now that I’ve said it, I probably will be less inclined to feel like I need to do it. And I think watching porn together will help too, because it’s more that sort of internalized like, that sort of shame around porn.

W: Well like, I had a lot of those thoughts when we were looking at whatever site, that anime furry site thing? I was like, ‘oh my God, he’s gunna feel disgusted by what I’m looking at’, or whatever.

(Winchester and Russell, partnered conversation)

Winchester and Russell’s conversation emphasized relational understandings of sex and desire and highlighted how culturally informed experiences of shame shaped how they negotiate sex in their relationship. By bringing their private fears into dialogue, this couple repositioned themselves in relation to sexual conservatism and created new space for partnered pleasure. They established further opportunities to negotiate partnered desire and pleasure by formulating the beginnings of a counter-script to structure future conversations about pornography.

R: We can just kind of like, introduce each other to the stuff that turns us on and, you know, likely some of it won’t, and maybe some that might be a surprise, but-

W: And then the other person could be like, “well you know, that’s cool, I’m not into that, but that’s totally wonderful that you enjoy that,” sort of thing.

R: Yeah, and I think it sort of like, would deepen our relationship and our intimacy…just to share that stuff with each other, you know?

(Winchester and Russell, partnered conversation)

This dialogical approach to sexual exploration challenged the cognitivist assumptions in which I was educated. These partners were not merely negotiating the divide between their internal and static sexual interests; rather, they were co-creating new possibilities for sex and pleasure through dialogue. By naming their own learned assumptions about the role of pornography in partnered sex, they further highlighted how broader cultural forces shape perceived sexual possibilities.

## Modifying consent through sexual scripting

Winchester and Russell similarly navigated learned assumptions when discussing kinky sex and consent. This tension arose as they discussed exploring a dominant/submissive sexual dynamic in which Russell initiates sex when he desires, whereas Winchester is positioned as available for sex at Russell’s choosing.

R: I also like the idea that you’re accessible, or you’re ready to be used whenever. And you make that known to me.

W: How do I make that known to you?

R: You tell me.

W: Oh okay, yeah.

R: Yeah, you tell me often that that’s what you like and that that makes you hot. And like, not that I didn’t believe it, but I’m like, acting on that more? And I will act on that more. Because like, I guess there’s part of me … that’s like, I would never want to do something that, you know … that you weren’t interested in or you weren’t in the mood [for].

(Winchester and Russell, partnered conversation)

Although both partners expressed desire to explore this dominant/submissive kink further, Russell shared reservations. A significant appeal of the kink is that sexual initiation is seemingly not negotiated between partners, creating tension between sexual consent and sexual pleasure. By fully stepping into the dominant sexual role, Russell expressed concern that he would violate his responsibility to attain consent from his partner, potentially pressuring Winchester into engaging in unwanted sex.

I would never want to do something to hurt somebody, or that they weren’t interested in, or that they weren’t in the mood for. And I wouldn’t want him to go through the motions just to please me.

(Russell, video-recall with researcher)

Winchester and Russell creatively modified culturally sanctioned consent scripts to ensure that their dominant/submissive sex was consensual and mutually pleasurable, without completely dismantling the power dynamic that makes the kink enjoyable. Instead of attaining verbal consent prior to sexual initiation, the couple agreed that consent is implied unless Winchester states otherwise. This restores a more balanced power dynamic in this kink; although when in play, Russell appears to have control over sexual initiation, both partners understand that Winchester is able and encouraged to revoke consent at his choosing.

This modified consent script thus requires Winchester to interrupt and decline undesired sexual advances. Winchester and Russell explored complex personal processes that make declining sex challenging.

R: Yeah, like I would like you to please be able to tell me, and vice versa

W: Yeah, absolutely.

R: You know, but that’s not sort of the most comfortable thing, because to initiate something, or like, if one person’s turned on and the other person isn’t into it-

W: Yeah, then that’s sort of awkward.

R: Then that’s sort of like, I don’t know, it’s sort of I guess a fear of rejection thing or something.

W: Yeah, or fear of the other person being upset that they’ve been rejected.

R: Yeah, or “why don’t you want me,” or, you know how we can get into our heads. I mean, well I can get into my head.

W: Oh yeah, absolutely, me too.

R: So permission to be like, “yeah, you know like, not right now,” or “you know, wait a little while,” or anything is absolutely okay. That’s actually, that’s fine with me. ‘Cause I would rather, than going through the motions with something.

(Winchester and Russell, partnered conversation)

Acknowledging that their modified approach to consent might elicit feelings of rejection, Russell offered alternative phrases the couple could use to mitigate potential hurt. “Not right now” and “you know, wait a little while” are presented as caring phrases to affectionately decline sexual initiation.

Mindful of personal sensitivities, interpersonal desires, and responsibilities, Winchester and Russell scripted an approach to kink that respected consent and pleasure. Again, the intricacies of this negotiation challenged comparatively fixed approaches to the psychological study of sex. Similar to how they co-created novel opportunities to integrate pornography into their partnered sex, this couple jointly constructed a kinky dynamic that was neither internally nor entirely culturally produced. By observing and analyzing partnered conversations and individual video-recall interviews, I was better able to appreciate these complex dialogical processes that challenge rigid and individualistic understandings of sex.

## Ryan and Greg: Observing interpersonal dynamics

Unlike Winchester and Russell, Ryan and Greg were less aligned in their views towards sexual exploration. During their partnered conversation, this couple discussed the possibility of introducing additional partners into their sexual relationship. Greg approached the conversation expressing a general disinterest in non-monogamy, whereas Ryan endorsed exploring extradyadic sex as a “fun” physical activity that could be separated from love. Qualitative video-recall procedures’ multiple data sources provided invaluable opportunities to observe how interpersonal dynamics shaped these conversations about sex.

During their partnered conversation, Ryan and Greg engaged in numerous negotiation strategies to reconcile their contrasting views towards monogamy. They presented more agreeable versions of themselves, with Ryan emphasizing that extradyadic sex would be meaningless fun and Greg expressing potential interest in exploring non-monogamy, should certain conditions be met. Speaking to one another’s interests served to establish commonality in a conversation characterized by difference.

G: I personally would have some limitations and boundaries, if we were to join another couple, or if someone were to join us.

R: I know you have limitations and boundaries. Like, you wouldn’t-

G: Like, I don’t feel comfortable…with like, kissing other guys? Which you-

R: And that’s not something I’m interested in at all. Like, those are reserved for you. But like, I don’t know, ‘cause [mutual friend] and his boyfriend have like…made it very clear that if we did ever want to do anything with them…it doesn’t have to be…necessarily us all going crazy, it could just be getting off in the same room? It doesn’t even have to [be] sexual, but like…does *that-…*if we were the two couples in the same room?

G: Yeah, that would-

R: Like, that’s something you’re totally comfortable with. But if-, yeah, I shouldn’t put words in your mouth, but it’s something you’re comfortable with.

G: No, definitely that is.

(Ryan and Greg, partnered conversation)

In contrast to this unifying approach to conversation, Ryan and Greg expressed more disparate viewpoints during video-recall. While engaging in guided reflections, they questioned whether their partner approached their conversation in a manner meant to please them.

He’d be like, “yeah, sure” [referring to engaging in extradyadic sex while on vacation]. Assuming, to my understanding anyways, that it would just never happen. So it’s very easy to say, “yeah, I’ll agree with you. I’ll be very agreeable”.

(Ryan, video-recall with researcher)

I think that he’s afraid if he were to say what he actually felt about the situation or wanted, that it might hurt me. But I don’t know, he can say whatever he wants. Like, if you want something, you can share that. Just be honest.

(Greg, video-recall with researcher)

By observing Ryan and Greg’s partnered conversations and separate video-recall interviews, I could readily explore how interpersonal dynamics shaped the content and processes of this couple’s dyadic communication. Consider how Greg discusses the possibility of exploring extradyadic sex differently when speaking with Ryan than when speaking with me.

Sometimes there is this occasional feeling where, yeah, sure let’s try and work out a way and branch out and see if we can bring someone in and join us, or try something new and include someone else in our sexual experiences. Uhh, but more so I’m leaning towards, like I am really comfortable and really content with if it were just to be us.

(Greg, partnered conversation with Ryan)

I want to tell him that…I don’t really mind if that happens or not… Whereas it’s him that is really encouraging this for us. Umm, I…kinda just wanna say that I don’t really care if anything happens with anyone.

(Greg, video-recall interview with researcher)

Greg’s varied perspectives enabled me to better appreciate the importance of interpersonal dynamics when studying partnered talk. Sexual communication involves continued negotiations and considerations of audience, as conversational partners hold significant power in how they interpret partners’ sexual disclosures. As Foucault states, “one does not confess without the presence (or virtual presence) of a partner who is not simply the interlocutor but the authority who requires the confession, prescribes and appreciates it, and intervenes in order to judge, punish, forgive, console, and reconcile” (Foucault, 1978: 61–62).

As gay men openly discussing sex in an academic setting, many relevant cultural forces circulated throughout Greg’s partnered conversation and video-recall interview. And although these forces undoubtedly warrant analytic consideration, qualitative video-recall procedures provided unique opportunities to also attend to interpersonal differences between conversational partners. Ryan and I held distinct positions when in conversation with Greg: that of a partner with whom he is deeply invested and that of a researcher whose position is in many ways tied to power and authority. Observing how our differing positions elicited nuanced responses, I became better able to appreciate how interpersonal dynamics shape sexual communication.

These reflections have encouraged me to further consider how power permeates qualitative sex research more broadly (Kong et al., 2001). As an interviewer who is at once shaped by the field of psychology, immersion in gay male culture, sex-positivity, queer teachings, sexology, and Whiteness (to name a few), I remain curious of how Greg and the other men in this study encountered me and how these interactions influenced the knowledge we produced. Despite our inability to step completely out of power (Foucault, 1978), I believe video-recall of more conventional qualitative interviews could provide novel vantage points from which to observe power and positionality in sex research. This could enable the field to more fully consider the challenges and possibilities of qualitative interviewing and could potentially inspire further innovations in this area of study.

## O and K: Deepening cultural reflexivity

Qualitative video-recall procedures’ collaborative and participatory approach also helped me honour participants’ unique experiences as distinct from my own. As with other approaches to interpretive inquiry, this pilot project’s qualitative text was analyzed through my culturally-informed discursive lens and the knowledge produced was shaped by my extant experience and knowledge (Holloway and Biley, 2011). As a gay male researcher conducting research with gay men, I was aware that my own experience and knowledge would shape my interpretive processes, and was mindful of the danger that my own perspectives could over-ride, misrepresent, or silence these participants’ distinct perspectives (Dwyer and Buckle, 2009). The dialogical and iterative processes used in qualitative video-recall procedures provided opportunities to critically reflect on how my interpretive voice might misrepresent participants’ perspectives (LaSala, 2003).

Consider the following excerpt from a conversation between O and K, a married gay couple from Indonesia. These men reflected on introducing threesomes into their sexual relationship during their partnered conversation. In the excerpt that follows, O attempted to normalize their threesome practices by drawing from the experiences of partnered gay men more broadly.

O: But again, since…I think it happens in most of gay men, I think, like…

K: I don’t know. I haven’t read any research about that, so I’m not going to jump to conclusion…

O: You have read a research…

K: I only read…I don’t know if like, most of gay men doing this.

(O and K, partnered conversation)

I initially read this section of conversation as K rejecting O’s desire to understand their experience by situating it in dominant, homonormative understandings. I believed that he was disinterested in whether “most” gay men were engaging in threesomes, instead preferring that the couple attend to their own unique experience. However, after reviewing the couple’s video-recall data, I came to appreciate how my own perspective distorted my understanding of K’s protest.

We always have this argument. I am a person who always reads something first, see the facts, read the numbers, statistics. And I can say, “okay, this is the majority of gay men is doing this”. So even though that there’s an article saying that a lot of gay people doing threesome, I still don’t believe that. I need to see the facts, what’s the number. Meanwhile, for me, [O] tend to say a lot of gay men right away, without saying the numbers. Like, assuming something. Like, just by seeing only three gay couple doing threesome, and he will say all of the gay people doing it.

(K, video-recall interview with researcher)

K’s frustration was not rooted in O’s comparison to dominant gay experiences. Contrarily, what I had initially read as K mocking O’s objective approach to understanding their subjective experience appeared instead to be K critiquing the credibility of O’s “facts.” For K, adherence to “facts” was highly valued, perhaps even more so than for his partner. Attending to participants’ interpretation of their own interaction provided alternative meanings that heightened researcher reflexivity.

The iterative interpretation process of analyzing video-recall data also helped surface cultural assumptions about affect and interpersonal communication dynamics. Socialized in a Western context, I initially experienced several of O and K’s communication dynamics and utterances as contemptuous. Their partnered conversation was characterized by crosstalk, exacerbated sighs, eye rolls, and forceful non-verbal gestures. They would often tell each other to “shut up” and were seemingly in constant battle over control of the conversation.

Despite initially reading this emotional intensity as hostility, I also experienced their conversation as highly affectionate. They verbally expressed their love for one another and offered affectionate gestures, including a kiss. Given the couple’s tempestuous dynamic, I reconsidered partners’ behaviors as playful teasing, instead of malicious. I explored this hunch further during my video-recall interview with K. In the following excerpt, I inquire about how K experienced his partner seemingly dismissing him in conversation.

MG: O responded with, “yeah, whatever”. I’m wondering how that felt for you to get that response.

K: Totally, sincerely, truthfully saying, nothing.

MG: Nothing.

K: It’s because like, me and O open all the boundaries and there’s no boundaries at all, so let it in, bring it on… we respect each other, reciprocally in the relationship… I know that if I’m opening myself to him, he will give me love. And if he opens himself to me, I will give him love too.

MG: So you can say things like, “yeah, whatever”.

K: Yeah, whatever, yeah.

MG: Because that’s authentic and true.

K: Exactly. And I can say to him, like, shut up.

(K, video-recall interview with researcher)

K discussed how behaviors that might be viewed as contemptuous in Western societies are highly valued in this couple’s relationship. He explained how expressing genuine and unfiltered emotion, regardless of its valence, facilitates closeness and trust in their relationship. K connected his blunt communication dynamic to his Indigenous Sumatran heritage and to his immersion in French culture and explained that his partner’s impassioned affect has been shaped by his involvement in queer advocacy in Indonesia.

Through engaging in exploratory video-recall and reflexive research practices, I could more easily identify when my own culturally learned assumptions unjustly colored how I perceived couples’ communication dynamics. Reflexive practices such as these are critical in discerning unique queer experiences that persist amidst neoliberalism’s continued infringement on queer life (Duggan, 2002). Rights-oriented movements of the twenty-first century have advanced hegemonic and depoliticized gay sexualities (Seidman, 2002), which Lisa Duggan discusses as homonormativity. Given homonormativity’s presumed Whiteness (Duggan, 2002), researchers entrenched in this normalizing discourse might fail to acknowledge important ethnoracial or cultural differences that inform many gay men’s sexual communication. Engaging in the process of video-recall has proven to be an invaluable tool in reflexively grappling with concerns of intersectionality.

## Considerations and future directions

Future research using qualitative video-recall procedures would invaluably advance empirical understandings of sexual communication. Although this pilot project’s sample represented many diverse intersections of identity, all couples were at least peripherally connected to a major metropolitan gay male community and were relatively experienced and comfortable with sexual communication. In addition to attaining larger sample sizes, further efforts are required to understand how sexual communication differs in less inclusive communities and between less communicative couples.

Despite these sampling limitations, the current pilot study elicited invaluable teachings in the ongoing effort to understand partnered sexual communication. In addition to enhancing my own learning and reflexivity, engaging in video-recall also provided participants with empowering opportunities for self-reflection. For example, Winchester engaged in a deeply personal reflective process that fortified his felt sense of safety in his relationship. By observing how he and Russell navigated anticipated feelings of hurt and rejection associated with declining sexual initiation, Winchester came to more fully appreciate the respect and care that he and his partner shared.

The place that I come from is one of very low self-worth. And so the belief that if someone was interested in me, in any way, that I didn’t have the right to reject them…because then they would leave, basically. They wouldn’t be interested in me…because I wasn’t inherently worthy, in that sense. So just the fact that we’re able to sit here and talk about that feeling of rejection and reassure one another that like, that’s okay? That’s huge…to have this discussion and have it be true…it’s not just me saying it, it’s actually like, this feeling of safety…to say no. And that’s okay. And to have Russell say no, and that’s okay.

(Winchester, video-recall with researcher)

By engaging in reflective video-recall processes, Winchester came into contact with an empowering realization of personal and partnered growth. This added benefit is noteworthy for helping professionals and those committed to emancipatory research practices. By engaging in these research procedures alongside compassionate others trained in empathic interviewing practices, participants can experience unique opportunities for self-reflection. Although the participants in this pilot project reported empowering and informative benefits from engaging in these practices, researchers should mindfully prepare for potential challenges and discomforts that this reflective process might also evoke.

The transformative experiences that I and my participants shared demonstrate personal and empirical benefits of using qualitative video-recall procedures in the areas of sex and sexuality. The dialogical and iterative joint interpretation processes resulted in culturally situated understandings of the interpersonal patterns queer men bring to their partnered sexual communication. Further application of qualitative video-recall procedures would elicit greater understandings of how couples navigate paradoxical social, political, and legal terrain in their sexual communication.

In this pursuit, we encourage researchers to use a variety of analytic conventions to advance observational understandings of partnered sexual communication. Although the current pilot study was guided by sexual script theory, additional discursive analyses, such as conversation analysis and Foucauldian discourse analysis, would further understandings of dialogical and sociopolitical factors that inform partnered sexual communication. By applying these analytic strategies to the rich data produced via qualitative video-recall procedures, researchers can more fully attend to the complex discursive terrain in which sexual communication is negotiated.
